# The ALPL gene variant project: results of the first 100 reclassified variants

**DOI:** 10.1093/jbmrpl/ziaf044

**Published:** 2025-03-17

**Authors:** Mariam R Farman, Theodora Malli, Catherine Rehder, Gerald Webersinke, Cheryl Rockman-Greenberg, Kathryn Dahir, Gabriel Á Martos-Moreno, Agnès Linglart, Keiichi Ozono, Lothar Seefried, Guillermo del Angel, Francesca Barbazza, Sara Shojaei, Jessica Ebner-Jahn, Florian Högler, Erica B Nading, Erin Huggins, Eric T Rush, Ahmed El-Gazzar, Josephine T Tauer, Priya S Kishnani, Wolfgang Högler

**Affiliations:** Department of Pediatrics and Adolescent Medicine, Johannes Kepler University Linz, Krankenhausstrasse 26-30, 4020 Linz, Austria; Laboratory for Molecular Genetic Diagnostics, Ordensklinikum Linz, Seilerstätte 4, 4010 Linz, Austria; Division of Medical Genetics, Department of Pediatrics, Duke University Medical Center, Durham NC 27705, United States; Laboratory for Molecular Genetic Diagnostics, Ordensklinikum Linz, Seilerstätte 4, 4010 Linz, Austria; Department of Pediatrics and Child Health, Rady Faculty of Health Sciences, University of Manitoba, Winnipeg R3a 1s1, Canada; Vanderbilt University Medical Center, Program for Metabolic Bone Disorders, Nashville, TN 37232, United States; Departments of Pediatrics & Pediatric Endocrinology, Hospital Infantil Universitario Niño Jesús, IIS La Princesa, 28009 Madrid, Spain; Department of Pediatrics, Universidad Autónoma de Madrid, 28029 Madrid, Spain; CIBER Fisiopatología de la Obesidad y Nutrición. ISCIII, 28029 Madrid, Spain; Paris Saclay University, INSERM, AP-HP, Bicêtre Paris-Saclay Hospital, 94270 Le Kremlin-Bicêtre, France; ISEIKAI International General Hospital, 530-0052 Osaka, Japan; Julius-Maximilian University, 97074 Würzburg, Germany; Centre for Genomics Research, Discovery Sciences, BioPharmaceuticals R&D, AstraZeneca, Boston, MA 02210, United States; Department of Pediatrics and Adolescent Medicine, Johannes Kepler University Linz, Krankenhausstrasse 26-30, 4020 Linz, Austria; Department of Pediatrics and Adolescent Medicine, Johannes Kepler University Linz, Krankenhausstrasse 26-30, 4020 Linz, Austria; Department of Pediatrics and Adolescent Medicine, Johannes Kepler University Linz, Krankenhausstrasse 26-30, 4020 Linz, Austria; Department of Pediatrics and Adolescent Medicine, Johannes Kepler University Linz, Krankenhausstrasse 26-30, 4020 Linz, Austria; Division of Medical Genetics, Department of Pediatrics, Duke University Medical Center, Durham NC 27705, United States; Division of Medical Genetics, Department of Pediatrics, Duke University Medical Center, Durham NC 27705, United States; Division of Clinical Genetics, Children's Mercy Hospital Kansas City, Kansas City, MO 64108, United States; Department of Internal Medicine, University of Kansas School of Medicine, Kansas City, KS 66103, United States; Department of Pediatrics, University of Missouri – Kansas City School of Medicine, Kansas City, MO 64108, United States; Department of Pediatrics and Adolescent Medicine, Johannes Kepler University Linz, Krankenhausstrasse 26-30, 4020 Linz, Austria; Division of Pulmonology, Department of Internal Medicine II, Medical University of Vienna, Comprehensive Cancer Center Vienna, 1090 Vienna, Austria; Department of Pediatrics and Adolescent Medicine, Johannes Kepler University Linz, Krankenhausstrasse 26-30, 4020 Linz, Austria; Division of Medical Genetics, Department of Pediatrics, Duke University Medical Center, Durham NC 27705, United States; Department of Pediatrics and Adolescent Medicine, Johannes Kepler University Linz, Krankenhausstrasse 26-30, 4020 Linz, Austria; Department of Pediatrics and Adolescent Medicine, Centre for Growth and Osteology, Kepler University Hospital Linz, 4020 Linz, Austria

**Keywords:** hypophosphatasia, alkaline phosphatase, vitamin b6, reclassification, ACMG, rickets

## Abstract

Hypophosphatasia (HPP) is an inherited disorder that affects bone and tooth mineralization, among other body systems. HPP is caused by pathogenic variants in the alkaline phosphatase-liver (*ALPL*) gene, which encodes tissue nonspecific alkaline phosphatase. One major challenge in diagnosing HPP is interpreting variant of uncertain significance (VUS), which can create uncertainty for patients and healthcare professionals, leading to delays in diagnosis and treatment or incorrect diagnoses. Since February 2021, the *ALPL*gene variant consortium has reclassified 100 VUS using adjusted American College of Medical Genetics/Association for Molecular Pathology criteria. Of these, 11 were reclassified as pathogenic, 62 as likely pathogenic, three as likely benign, one as benign, and 23 remained as VUS; out of 100 variants, there were 81 missense, eight frameshifts, four in-frame deletions, two intronic, two synonymous, two nonsense, and one splice site variant. *In vitro*functional testing of the variant’s residual enzymatic activity and its dominant negative effect (DNE) was required for 40 variants**.**Out of 40 variants tested, 72% showed <30% residual activity and were reclassified as pathogenic or likely pathogenic. Among these, 48% exhibited a DNE. All variants with ˂15% residual activity showed a DNE, while those with over 20% did not. In terms of domains, all tested variants in the active site domain and 75% of those in the homodimeric interface showed <30% residual activity. The consortium’s reclassification of 100 *ALPL*variants has resulted in a 21% increase in the number of variants in the Johannes Kepler University *ALPL*gene variant database. Furthermore, 136 new genotypes with 118 associated phenotypes, including asymptomatic states, have been added. These expanded resources will help improve genetic counseling for patients and families affected by *ALPL*variants and enable faster diagnosis and medical decision-making.

## Introduction

Hypophosphatasia (HPP) is a rare polymorphic metabolic disorder primarily affecting bone and dental mineralization but also exhibiting nonskeletal manifestations. HPP results from monoallelic or biallelic mutations in the alkaline phosphatase-liver (*ALPL*) gene, leading to a deficiency or dysfunction of tissue-nonspecific alkaline phosphatase (TNSALP).^1^TNSALP deficiency is reflected in low alkaline phosphatase (ALP) activity. The *ALPL*gene, located on human chromosome 1p36.12,^2^comprises 12 exons and spans ˃70 kb in size.^3^*ALPL*encodes the TNSALP protein comprising 524 amino acids, which acts as a homodimeric enzyme facilitating the hydrolysis of phosphate-containing molecules outside the cells, thereby regulating hydroxyapatite deposition in the extracellular matrix.^4^Additionally, homodimeric TNSALPs can tetramerize into an octamer (containing four homodimers or eight *ALPL*transcripts), hypothesized to stabilize TNSALP functions in extracellular environments.^4^

Clinical manifestations of HPP exhibit multi-organ involvement and affected patients display the biochemical signature of HPP, comprising low serum levels of ALP for age and sex and accumulation of TNSALP substrates such as pyridoxal 5′-phosphate, inorganic pyrophosphate, and phosphoethanolamine.^5^Symptoms of HPP include, among others, rickets-like bone changes, bone hypomineralization, osteomalacic pseudofractures, muscle weakness, musculoskeletal pain, early tooth loss, craniosynostosis, nephrocalcinosis, and chondrocalcinosis.^6–9^Untreated neonates and infants have the worst prognosis due to respiratory insufficiency with chest deformity and pulmonary hypoplasia, vitamin B6-responsive seizures, and a high rate of complications.^10^Manifestations of HPP have traditionally been classified into six phenotypes: prenatal benign, perinatal lethal, infantile, early and late childhood, adult, and odonto HPP, based on severity and age of first symptoms.^11^^,^^12^Subtypes show symptomatic overlap and do not take the longitudinal course of disease into account. HPP can be inherited in an autosomal dominant (AD) or autosomal recessive (AR) manner, with the more severe end of the spectrum typically attributed to AR disease. Heterozygous individuals are either asymptomatic carriers or have dominantly inherited HPP. Such AD HPP is often caused by variants with a dominant negative effect (DNE) where the mutant protein disrupts the function of the WT protein; however, in some AD HPP cases without apparent DNE, the mechanism of disease is unclear. These broad traditional HPP forms do not accurately reflect the phenotypic inter- and intraindividual variability, which complicates and delays HPP diagnosis and treatment.^12^^,^^13^Several other factors also delay the diagnosis of HPP, including limited knowledge and awareness of HPP in the medical community, incomplete penetrance of dominant forms, and variable expression within and between families.^14^To date, several algorithms have been published to aid in diagnosing HPP.^15–17^Considering the heterogeneous clinical manifestations and progressive nature of the disease,^7^^,^^14^achieving a correct and precise diagnosis, especially in attenuated cases, presents a significant challenge.^18^^,^^19^

A timely, accurate diagnosis of HPP is essential to access specialist health care including access, if indicated, to the only causative treatment available which is enzyme replacement therapy (ERT) with asfotase alfa.^20^

Due to the impracticality of covering every rare disease in the medical school curriculum, there is a widespread deficiency in healthcare professionals’ knowledge regarding the clinical presentation, and biochemical signature of HPP. Milder or less specific signs and symptoms will lead to delayed referrals to bone specialists. However, even specialists may encounter difficulties that require assistance in making a correct diagnosis, particularly since low serum ALP is quite common^7^^,^^16^^,^^21^^,^^22^in many conditions, so genetic confirmation helps. In a significant number of cases, genetic testing uncovers a variant of uncertain significance (VUS) in the *ALPL*gene, complicating the diagnostic process. This can lead to challenges in determining the precise implications of the genetic variation, requiring further investigation and analysis for accurate interpretation and subsequent clinical decision-making. Thus, the *ALPL*gene variant project is a crucial initiative focused on reclassifying VUS and consistently evaluating and revising variant-specific data, including associated genotypic, phenotypic, and functional information in the Johannes Kepler University (JKU) *ALPL*gene variant database (https://ALPLmutationdatabase.jku.at/), which serves as an openly accessible repository for the global medical community. Here, we present the results of the first 100 reclassified variants. We also assess the correlation between variant pathogenicity and residual activity, type of variant, and variant location within the protein domains.

## Materials and methods

### Data collection & type of study

The 100 *ALPL*variants originated from three different sources: first, a significant portion of variants (42/100), initially labeled as “unknown pathogenicity,” was acquired in 2021 when our team took over the SESEP database curated by Prof. Etienne Mornet during his time at the University of Versailles-Saint Quentin en Yvelines, France. Second, VUSs (25/100) were submitted through the project’s submission portal (https://alplmutationdatabase.jku.at/portal/). All remaining VUSs (33/100) were identified through deep literature mining or reported personally by colleagues.

Patients/legal guardians provided informed consent to submit the clinical details for the variants through the project’s submission portal. Ethics approvals were obtained, as previously reported.^17^

### Variant classification process

**Figure 1:**
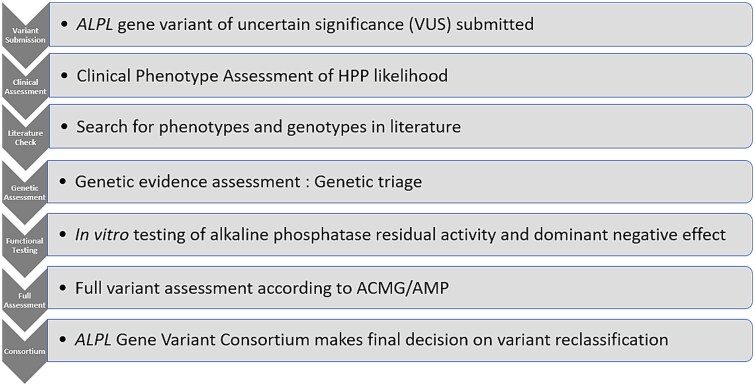
Flowchart illustrating the seven distinct steps of the variant classification process.

The established international multidisciplinary consortium of HPP experts undertook variant classification or reclassification through a meticulous multi-step process based on the classification guidelines set forth by the American College of Medical Genetics and Genomics (ACMG) and the Association for Molecular Pathology (AMP),^23^with some adaptations for the *ALPL*gene and HPP (see Table S1). This process includes evaluating clinical phenotypes, conducting a comprehensive literature review utilizing advanced artificial intelligenc (AI) search software Mastermind,^24^genetic assessment, *in vitro*functional testing (when necessary and feasible) and consortium review. A detailed description of all processes has been recently published^17^; however, a brief overview of the process involved is depicted in Figure 1.

### *In vitro*functional testing

**Figure 2:**
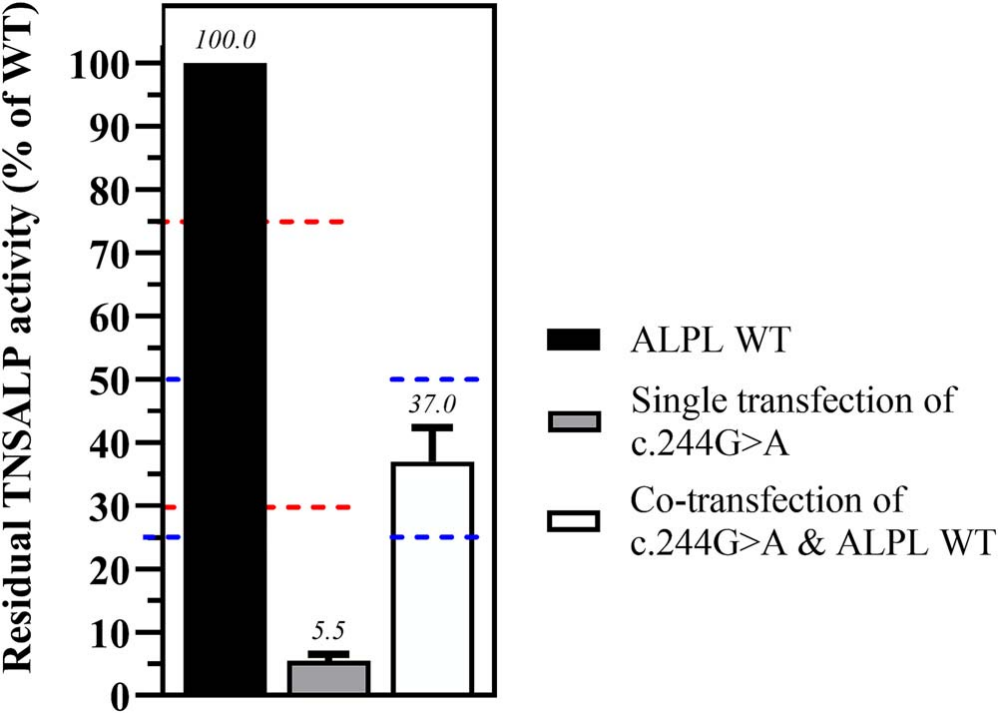
*In vitro*functional testing output for the *ALPL*variant c.244G>A, categorized as likely pathogenic and dominant negative. The bar graph represents the average residual enzymatic activity of three independent replicates per transfection (mean +/− SD). The left bar represents the transfection with the *ALPL*WT plasmid, while the middle bar represents the single transfection with the *ALPL*c.244G>A-mutant plasmid. The right bar depicts the co-transfection of the WT and mutant plasmid at a ratio of 50:50. The residual activity of the mutant plasmid and co-transfection are normalized to the activity of the single WT transfection (=100%). The applied threshold for residual activity used for mutation categorization is indicated as a red dotted line (single transfection, residual activity threshold <30%; supportive of damaging effect on protein, PS3 criterion from ACMG applied in moderate strength for residual activity 30%-50%; and >75% for no damaging effect, BS3 from ACMG applied in moderate strength). In the given example, c.244G>A shows a residual TNSALP activity of 5.5% (middle bar, single transfection), which falls below the 30% threshold and therefore has a predicted damaging effect based on functional testing. When co-transfected with the WT plasmid, the mean residual activity is 37%, indicating that this variant has a dominant negative effect (DNE if residual activity 25%-50%).

The functional testing utilized the transfection of Madin-Darby Canine Kidney II cells (ECACC, #00062107) with mutant and/or wild-type (WT) *ALPL*and the detection of residual enzymatic TNSALP activity. The selected cells exhibit negligible endogenous ALP activity, which makes them suitable for studying the effects of transfected variants. For this, mutations were introduced into an *ALPL*plasmid via site-directed mutagenesis (Agilent, #200522) and confirmed by Sanger sequencing. Transfection (lipofectamine 3000, Invitrogen™, #L3000015) involved using 100% *ALPL*WT plasmid (Origene, #RC205692) as control, or 100% mutant plasmid (in the following referred to as “single transfection”), or a mix of 50% WT/50% mutant plasmid (in the following referred to as “co-transfection”). To standardize and normalize TNSALP residual activity, a β-galactosidase plasmid (Promega, #E108A) was co-transfected in all conditions. Specifically, for single transfections, 0.5 μg of the mutant plasmid and 0.5 μg of pSV-β-Galactosidase plasmid were used per well in a 24-well plate. For co-transfections, 0.25 μg of the mutant plasmid, 0.25 μg of WT plasmid, and 0.5 μg of pSV-β-Galactosidase plasmid were transfected per well in the same plate. Plasmid maps are available in the Supplement. After 48 hours, TNSALP and β-galactosidase activities are assessed. For that, transfected cells were harvested and lysed. Cell lysates were then incubated with β-galactosidase reagent (Thermo Scientific, #75705) or TNSALP substrate (obtained by dissolution of 1 tablet p-Nitrophenyl Phosphate [PNPP; Thermo Scientific, #34047] in 5 mL of Phosphatase Alkaline buffer), respectively. Absorbance readings at 405 nm for ALP are normalized against β-galactosidase. Each variant was tested in three independent experiments per transfection method. The average of these three independent experiments (mean +/− standard deviation) was used for further analysis. For additional methodological details, please see the previous publication.^17^An example of the functional testing output is depicted in Figure 2.

### TNSALP structure

**Figure 3:**
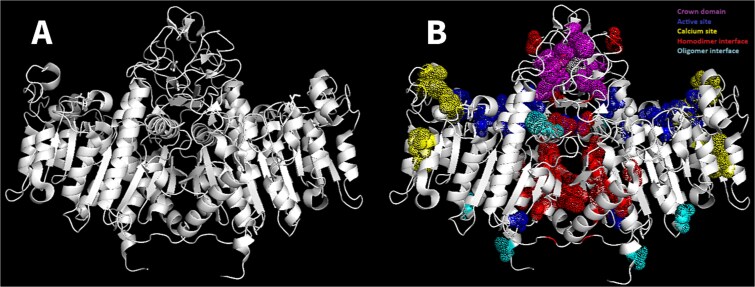
Ribbon 3D structure of a TNSALP homodimer. (A) 3D structure of the TNSALP homodimer. (B) Display of 52 out of 100 variants reclassified at specific amino acid residue locations within the active site (blue), crown domain (magenta), calcium site (yellow), homodimeric interface (red), and oligomer interface (cyan). The 48 variants, which are not displayed, are located in unspecified regions of the TNSALP protein. The variants are color-coded according to their respective domains for both pairs of the homodimer.

The structure of TNSALP is complex, featuring an X-shape homo-octamer formed by four TNSALP-homodimers stabilized by molecular interactions.^4^Figure 3Aillustrates the typical 3D structure of a single TNSALP homodimer. The 3D structure was generated using the PyMOL software,^25^utilizing the TNSALP PDB structure file obtained from the RCSB (7YIV) PDB database.^26^With PyMOL, mutated residues were identified and color-coded according to domains identified by Silvent,^4^^,^^27^including additional domains such as the signal peptide, ionic pocket (not shown), and oligomer interface^4^shown in Figure 3B.

### Statistics

If not indicated differently, data (residual TNSALP activity, % of WT) are presented as mean +/− standard deviation. Graphically, data are presented either as scatter plots showing group mean (straight black line), along with individual samples included in the analysis, or as box plots, depicting the group median (black line), mean (+), minimum and maximum, and each variant, respectively. Unpaired *t*-tests, nonlinear regression analyses and Pearson correlations were used for statistical analysis. A *p*-value below 0.05 was considered statistically significant. Statistical analysis and graphical illustration were generated using GraphPad Prism software (Version 10.2.2.).

## Results

### Reclassification of 100 variants of uncertain significance

Since its inception in February 2021, the ALPL gene variant consortium has reclassified 100 *ALPL*variants (Table 1). Forty-two variants were reclassified, which were stated as of “unknown pathogenicity” in the data transferred from the SESEP database; 25 variants came from submissions to the JKU ALPL gene variant database, and 33 variants were extracted from literature mining. All 100 variants underwent extensive literature mining, resulting in 60 variants being reclassified without need for additional *in vitro*functional testing since sufficient evidence was found to allow reclassification. The remaining 40 VUSs required further functional testing for reclassification. All successfully reclassified variants were added to the JKU ALPL gene variant database and were submitted to ClinVar.^28^

**Table 1:** List of 100 reclassified *ALPL*variants.

Variant (Base change)	Location	Mutation type	Classification	ACMG/AMP applied
**c.41T>C**	Exon 2	Missense	LP	PS3_Mod, PM2_sup, PM3_sup, PP2_sup, PP4_sup, PP3_sup
**c.62-29G>A**	IVS3	Intronic	LB	PM2_sup, BP2_sup, BP7_sup
**c.69_74del**	Exon 3	In-frame deletion	LP	PS3_mod, PM4_mod, PM2_sup, PP4_sup
**c.106A>C**	Exon 3	Missense	LP	PS3_Mod, PM2_sup, PM3_sup, PP2_sup, PP4_sup, PP3_sup
**c.140A>T**	Exon 3	Missense	LP	PS3_mod, PP4_mod, PM2_sup, PM3_sup, PP2_sup, PP3_sup
**c.146A>T**	Exon 3	Missense	LP	PS3_mod, PM2_sup, PP2_sup, PP4_sup, PP3_sup
**c.176G>A**	Exon 3	Missense	VUS	PM2_sup, PP2_sup, PP4_sup
**c.178G>C**	Exon 3	Missense	LP	PM1_mod, PS3_sup, PM2_sup, PP2_sup, PP3_sup, PP4_sup
**c.182-2A> G**	IVS3	Splice site	LP	PVS1_Strong, PM2_sup, PP4_sup
**c.194C>A**	Exon 4	Missense	LP	PS3_mod, PP4_mod, PM2_sup, PM3_sup, PP2_sup, PP3_sup
**c.206C>T**	Exon 4	Missense	VUS	PM2_sup, PP2_sup, PP3_sup
**c.214A>G**	Exon 4	Missense	LP	PS3_mod, PP4_mod, PM2_sup, PM3_sup, PP2_Sup,, PP3_sup
**c.237_238delCA**	Exon 4	Frameshift	LP	PVS1, PM2_sup
**c.244G>A**	Exon 4	Missense	LP	PM2_sup, PP2_sup, PP3_sup, PP4_sup, PS3_sup, PP1_sup
**c.244G>C**	Exon 4	Missense	LP	PS1_mod, PM2_sup, PP1_sup, PP2_sup, PP3_sup, PP4_sup
**c.286G>C**	Exon 4	Missense	LP	PS3_mod, PM2_sup, PP4_sup, PP2_sup, PP3_sup
**c.295A>G**	Exon 4	Missense	LP	PS3_mod, PM2_sup, PP2_sup, PP3_sup, PP4_sup
**c.297+5G>A**	IVS4	Intronic	VUS	PM2_sup, PM3_sup, PP3, PP4_sup
**c.302A>G**	Exon 5	Missense	LP	PP4_mod, PS3_mod, PM2_sup, PP2_sup, PP3_sup,
**c.306C>T**	Exon 5	Synonymous	LB	PM2_sup, BP4_sup, BP7_sup
**c.319G>A**	Exon 5	Missense	LP	PP4_mod, PS3_mod, PM2_sup, PM3_sup, PP2,
**c.330T>C**	Exon 5	Synonymous	B	BA1
**c.361G>A**	Exon 5	Missense	LP	PP4_mod, PS3_sup, PM2_sup, PP2_sup, PP3_sup
**c.371A>G**	Exon 5	Missense	LP	PS3_mod, PM2_sup, PP2_sup, PP3_sup, PP4_sup
**c.410delC**	Exon 5	Frameshift	P	PVS1, PM2_sup, PP4_sup
**c.412dup**	Exon 5	Frameshift	P	PVS1, PP4_strong, PM2_sup
**c.431G>C**	Exon 5	Missense	VUS	PP2_sup, PM2_sup, PP3_sup
**c.433A>G**	Exon 5	Missense	VUS	PP2_sup, PM2_sup, PP4_sup
**c.466G>T**	Exon 5	Missense	VUS	PM2_sup, PP2_sup, PP3_sup, BS3_mod
**c.498_500del**	Exon 6	In-frame deletion	VUS	PM4_mod, PM2_sup
**c.511C>G**	Exon 6	Missense	LP	PM1_mod, PM3_mod, PM2_sup, PP2_sup, PP3_sup, PP4_sup
**c.527C>T**	Exon 6	Missense	LP	PM2_sup, PM3, PM5, PP2_sup, PP3, PP4_sup
**c.538C>A**	Exon 6	Missense	VUS	PM2_sup BS3_sup, PP3_sup
**c.539A>G**	Exon 6	Missense	VUS	PS3_mod, PP2, PM2_sup, PP3
**c.542C>T**	Exon 6	Missense	P	PP4_strong, PM3_mod, PS3_sup, PP2_sup, PM2_sup, PP3_sup
**c.550C>T**	Exon 6	Missense	P	PP4_strong, PM1_mod, PM2_sup, PP3_sup, PP2_sup, PS3_sup
**c.558G>A**	Exon 6	Nonsense	P	PVS1, PP4_sup, PM2_sup
**c.560A>G**	Exon 6	Missense	LP	PM2_sup, PM1_mod2, PP2_sup, PP3_sup, PP4_sup
**c.572A>G**	Exon 6	Missense	LP	PM3_mod, PM5_mod, PM2_sup, PP2_sup, PP3_sup, PP4_sup
**c.601T>C**	Exon 6	Missense	LP	PS3_mod, PM2_sup, PP3_sup, PP4_sup, PP2_sup
**c.613G>A**	Exon 6	Missense	VUS	PM2_sup, PP2_sup, PP3_sup
**c.625A>T**	Exon 6	Missense	VUS	PS3_sup, PM2_sup, PP4_sup, PP2_sup, PP3_sup
**c.649_650insC**	Exon 7	Frameshift	P	PVS1, PM3_mod, PM2_sup
**c.657G>T**	Exon 7	Missense	LP	PS3_mod, PP4_mod, PM2_sup, PP2_sup
**c.659G>C**	Exon 7	Missense	P	PP4_strong, PM3_strong, PS3_mod, PM2_sup, PP2, PP3
**c.659G>T**	Exon 7	Missense	LP	PM3_mod, PM2_sup, PP4_sup, PP2, PM5
**c.675_676insCA**	Exon 7	Frameshift	LP	PVS1, PM2_sup
**c.707A>G**	Exon 7	Missense	LP	PM3_mod, PS3_mod, PM2_sup, PP2_sup, PP3_sup
**c.715G>T**	Exon 7	Missense	VUS	PM2_sup, PM3_sup, PP2_sup, PP3_sup, PP1_sup, PS3_sup
**c.768G>A**	Exon 7	Nonsense	P	PVS1, PM2_sup, PP4_sup
**c.793-14_33del**	IVS7	In-frame deletion	LP	PP4_strong, PM2_sup, PP3_sup, PS3_sup
**c.802T>C**	Exon 8	Missense	LP	PS3_mod, PM3_mod, PM2_sup, PP2_sup, PP4_sup
**c.818C>T**	Exon 8	Missense	VUS	PP2_sup, PP4_sup, BP4, BS2_sup
**c.871G>C**	Exon 9	Missense	LP	PP4_mod, PM2_sup, PM3, PM5, PP2, PP3, PS3_sup
**c.906C>A**	Exon 9	Missense	VUS	PM2_sup, PP2_sup
**c.955A>T**	Exon 9	Missense	VUS	PM2_sup, PP2_sup, PP3_sup
**c.967A>G**	Exon 9	Missense	VUS	PM2_sup, PP2, PP3, PP4_sup, PS3_sup
**c.979T>C**	Exon 9	Missense	LP	PP4_strong, PM2_sup, PP2, PM3, PP3
**c.1000G>A**	Exon 10	Missense	LP	PP4_mod, PS3_mod, PM2_sup, PM3_sup, PM5, PP2_sup, PP3_sup
**c.1004G>C**	Exon 10	Missense	LP	PS3_mod, PM2_sup, PP4_sup, PP2_sup, PP3_sup
**c.1015G>A**	Exon 10	Missense	LP	PS3_mod, PP4_sup, PM2_sup, PP2_sup, PP3_sup
**c.1018C>T**	Exon 10	Missense	LP	PP4_strong, PM2_sup, PP2_sup, PP3_sup
**c.1034C>T**	Exon 10	Missense	LP	PP4_mod, PS3_mod, PM2_sup, PP2_sup, PP3
**c.1101_1103del**	Exon 10	In-frame deletion	P	PM3_strong, PP4_strong, PM2_sup, PM4_sup
**c.1120G>A**	Exon 10	Missense	LP	PM2_sup, PM3, PP2, PP3, PP4_sup, BS3
**c.1132G>T**	Exon 10	Missense	LP	PS3_mod, PM1, PM2_sup, PM5, PP2_supp, PP3
**c.1156G>C**	Exon 10	Missense	LP	PS3_mod, PM2_sup, PP2_sup, PP3_sup, PP4_sup
**c.1157G>A**	Exon 10	Missense	LP	PS3_mod, PM2_sup, PP2_sup, PP3_sup, PP4_sup
**c.1213A>C**	Exon 11	Missense	LP	PS3_mod, PM2_sup, PP2_sup, PP4_sup, PM3_sup
**c.1225C>G**	Exon 11	Missense	VUS	PM2_sup, PP2_sup, PP3_sup, PP4_sup
**c.1225C>T**	Exon 11	Missense	VUS	PM2_sup, PP2_sup, PP3_sup, PP4_sup, PS3_sup
**c.1243T >G**	Exon 11	Missense	LP	PP4_mod, PM2_sup, PM3_sup, PP2_sup, PP3_sup
**c.1247G>A**	Exon 11	Missense	LP	PS3_mod, PM2_sup, PP2_sup, PP3_sup, PP4_sup
**c.1247G>T**	Exon 11	Missense	LP	PP4_mod, PM2_sup, PP2_sup, PP3_sup, PM5_sup
**c.1258G>A**	Exon 11	Missense	LP	PS3_mod, PM2_sup, PM3, PP2, PP3, PP4_sup
**c.1259G>A**	Exon 11	Missense	LP	PS3_mod, PM2_sup, PM5_sup, PP2, PP3
**c.1259G>C**	Exon 11	Missense	LP	PS3_mod, PM2_sup, PM3_sup, PP2, PP3
**c.1283G>A**	Exon 11	Missense	LP	PS3_mod, PM2_sup, PM5, PP2_sup, PP3, PP4_sup
**c.1327G>A**	Exon 12	Missense	LP	PP4_mod, PS3_mod, PM2_sup, PM5, PP2
**c.1327G>T**	Exon 12	Missense	LP	PS3_mod, PM2_sup, PP2_sup, PM3_sup, PP4_sup
**c.1328C>G**	Exon 12	Missense	LP	PP4_strong, PM2_sup, PP2_sup, PM5_sup
**c.1331A>G**	Exon 12	Missense	LP	PS3_mod, PM2_sup, PP2_sup, PP3_sup, PP4_sup
**c.1333T>A**	Exon 12	Missense	VUS	PM2_sup, PP2_sup
**c.1366G>A**	Exon 12	Missense	LP	PP4_mod, PS3_mod, PM2_sup, PP2, PP3
**c.1367G>A**	Exon 12	Missense	LP	PS3_mod, PM2_sup, PM5_sup, PP2, PP3, PP4_sup
**c.1376T>C**	Exon 12	Missense	LP	PS3_mod, PM2_sup, PP4_sup, PP3, PP2
**c.1379C>T**	Exon 12	Missense	VUS	PM2_sup, PP2_sup, PP3_sup, PP4_sup
**c.1381G>A**	Exon 12	Missense	LB	PP4_sup, BS1, BP4, BS3_sup
**c.1402G>A**	Exon 12	Missense	P	PP4_strong, PS3_mod, PM2_sup, PM3_sup, PP2, PP3
**c.1403C>T**	Exon 12	Missense	LP	PS3_mod, PM2_sup, PM5, PP2, PP3, PP4_sup
**c.1415A>G**	Exon 12	Missense	LP	PM3_mod, PM2_sup, PP2_sup, PP3_sup, PP4_sup, PS3_sup
**c.1418G>A**	Exon 12	Missense	LP	PP4_mod, PM3_mod, PM5_sup, PP2_sup, PP3_sup, PM2_sup
**c.1436A>G**	Exon 12	Missense	LP	PP4_mod, PM2_sup, PM3_sup, PP2_sup, PP3_sup
**c.1444C>A**	Exon 12	Missense	LP	PP4_strong, PM2_sup, PM3, PP2, PP3
**c.1444C>T**	Exon 12	Missense	VUS	PM2_sup, PP2_sup, PP3_sup
**c.1444dup**	Exon 12	Frameshift	LP	PVS1_strong, PM3, PM2_sup, PP4_sup
**c.1483G>A**	Exon 12	Missense	VUS	PP2_sup, PM2_sup, PP4_sup, PM3_sup, BP4
**c.1487A>G**	Exon 12	Missense	VUS	PP4_mod, PM2_sup, PP2_sup
**c.1489T>A**	Exon 12	Missense	LP	PM2_sup, PM3, PP2, PP3, PP4_sup
**c.1532insC**	Exon 12	Frameshift	P	PVS1, PM2_sup, PM3_sup, PP4_sup

Abbrevations: B, benign; LB, likely benign; LP, likely pathogenic; P, pathogenic; VUS, variant of uncertain significance.

**Figure 4:**
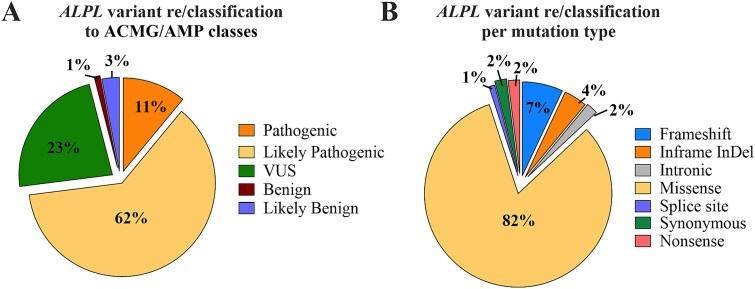
Reclassified *ALPL*variants by pathogenicity (A) and mutation type (B).

Data analysis of the 100 reclassified variants identified 11 as pathogenic, 62 as likely pathogenic, three as likely benign, and one as benign. Twenty-three variants remained VUS due to insufficient phenotypic data or inconsistencies between the phenotype and *in vitro*functional studies (Figure 4A). Regarding the type of variants, 82/100 of the reclassified variants were missense, seven were frameshift, four were in-frame indel, two were intronic, one was a splice site, two were synonymous, and two were nonsense variants (Figure 4B). All frameshift variants, in-frame indels, splice site alterations, and nonsense mutations were reclassified as pathogenic or likely pathogenic, underscoring the clinical significance of these mutations. Conversely, intronic and synonymous variants were categorized as benign or likely benign (Figure S1A). Analyzing the distribution of the 100 reclassified *ALPL*variants across the different exons of the ALPL gene revealed, the highest number (22%) of reclassified variants were found in exon 12. Variants in exons 10 and 4 almost exclusively were reclassified as pathogenic or likely pathogenic variants, with only one VUS in exon 4 (Figure S1B).

Next, we evaluated potential hotspots of variant accumulation within the TNSALP protein structure (Figure 3). Fifty-four reclassified variants were located in the previously described domains^4^^,^^27^: crown domain, active site, calcium site, homodimeric interface, signal peptide and oligomer interface of the TNSALP protein (Figure 3B**)**. The remaining reclassified variants were either located in an unknown domain (no functional domain), near the active site, or in an intramolecular stabilization site. Out of the 100 reclassified variants, the homodimeric interface domain (red, Figure 3B) contained one pathogenic, 20 likely pathogenic, three VUS and one likely benign variant. The active site (blue, Figure 3B) had one pathogenic and five likely pathogenic variants, while the calcium site (yellow, Figure 3B) had nine variants; one reclassified as pathogenic, four as likely pathogenic, and four remained VUS. The crown domain (magenta, Figure 3B) had six likely pathogenic and three VUS, while the oligomer interface (cyan, Figure 3B) had one each for likely pathogenic, pathogenic, and VUS. The signal peptide (not shown in Figure 3B) had two variants, both likely pathogenic.

### *In vitro*residual TNSALP activity is associated with pathogenicity and variant protein domain location

**Figure 5:**
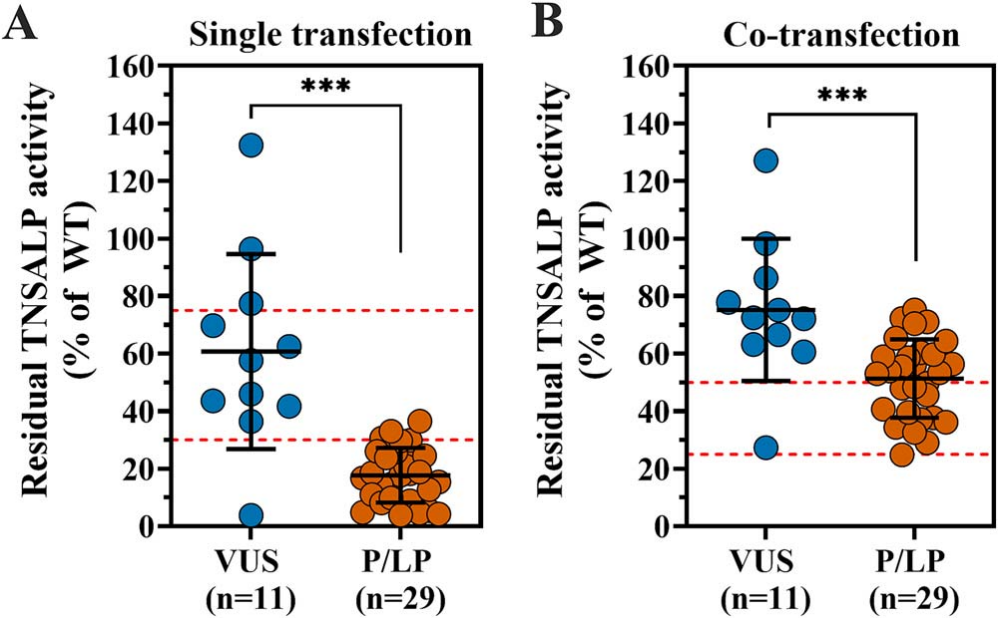
The fractions of WT-relative residual TNSALP activity based on single (A) and co-transfection model (B) for VUS (blue dots, *n* = 11) and pathogenic/likely pathogenic *ALPL*variants (orange dots, *n* = 29). The data are displayed as a dot plot, with the mean indicated by a straight black line, along with each sample included in the analysis. Red dotted lines indicate the 30 and 75% applied thresholds for single transfection, and the 25%-50 % DNE range for co-transfection. Statistics: unpaired *t*-test. ***, *P* < .001. Abbrevations: LP, likely pathogenic; P, pathogenic; TNSALP, tissue-nonspecific alkaline phosphatase; VUS, variant of uncertain significance.

Next, we analyzed the residual TNSALP activity of the 40 variants tested functionally *in vitro*. Table S2includes the variants tested at the JKU research laboratory (Linz, Austria) along with their corresponding pathogenicity, single transfection residual activity, and co-transfection residual activity. Specifically, after single transfection, 29 out of 40 variants exhibited residual enzymatic activity around or below the 30% cut-off, suggesting a damaging effect. Conversely, 11 variants demonstrated residual activity above 30% and are currently classified as VUS (Figure 5A). Additionally, 14 of the 29 variants reclassified as pathogenic/likely pathogenic were also dominant negative, as their residual activity was between 25% and 50% on co-transfection with WT enzyme. All reclassified dominant negative variants comprised missense variants, including five variants in the active site, four in the homodimeric region, two in the crown domain and three in unspecified regions. Variants that remained VUS showed residual activity above the 50% threshold in the co-transfection experiments, indicating they are not dominant negative (Figure 5B). One variant (c.176G>A) exhibited residual activity of 3.8% after single transfection and 27.3% after co-transfection, which suggests a damaging and DNE. However, a pathogenic/likely pathogenic classification could not be reached for this variant, due to a lack of phenotypic data.

**Figure 6:**
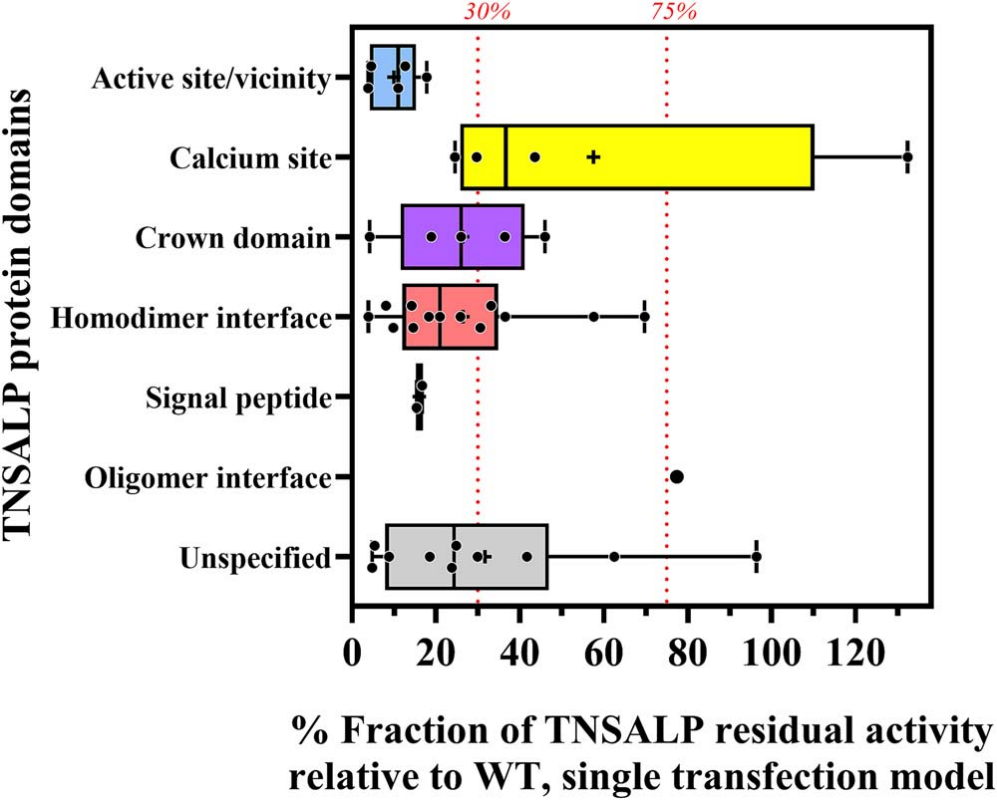
WT-relative *in vitro*residual TNSALP activity categorized by TNSALP protein domain. Data (*n* = 40) are presented as boxplots showing the median (black line), mean (+), minimum and maximum (whiskers), and each variant’s residual activity (single black dots). The red dotted lines represent the residual activity in a single transfection cut-off of 30% for pathogenic and 75% for benign variants. TNSALP protein domain structure was classified as described by Silvent et al. and Yu et al.,^4^^,^^22^and boxplots are color-coded as described in Figure 4.

Further analysis of missense/in-frame variant-related residual activity across known protein domains demonstrated that variants with low TNSALP residual activity (˂30%) were predominantly located in the active site or its vicinity, the crown domain, or the homodimeric interface (Figure 6). All variants tested *in vitro*from the active site domain (5 tested out of 6) showed single transfection residual activity below 30% and a DNE, and were reclassified as either pathogenic or likely pathogenic. The homodimeric interface had 25 variants, with 84% of them being reclassified as pathogenic or likely pathogenic (see Figure S1C). Out of the 12 variants from the homodimeric interface that were tested *in vitro*, 75% had a single transfection residual activity below 30%, and only 33% showed a DNE. In the crown domain, nine variants were reclassified, out of which five were tested *in vitro*; only three tested variants exhibited residual activity below 30%, and only one variant showed DNE. Four of the nine variants in the calcium site were tested *in vitro*; only one showed residual activity lower than 30% with no DNE. Variants in the signal peptide domain also exhibited low TNSALP residual activity, but did not demonstrate dominant negativity.

**Figure 7:**
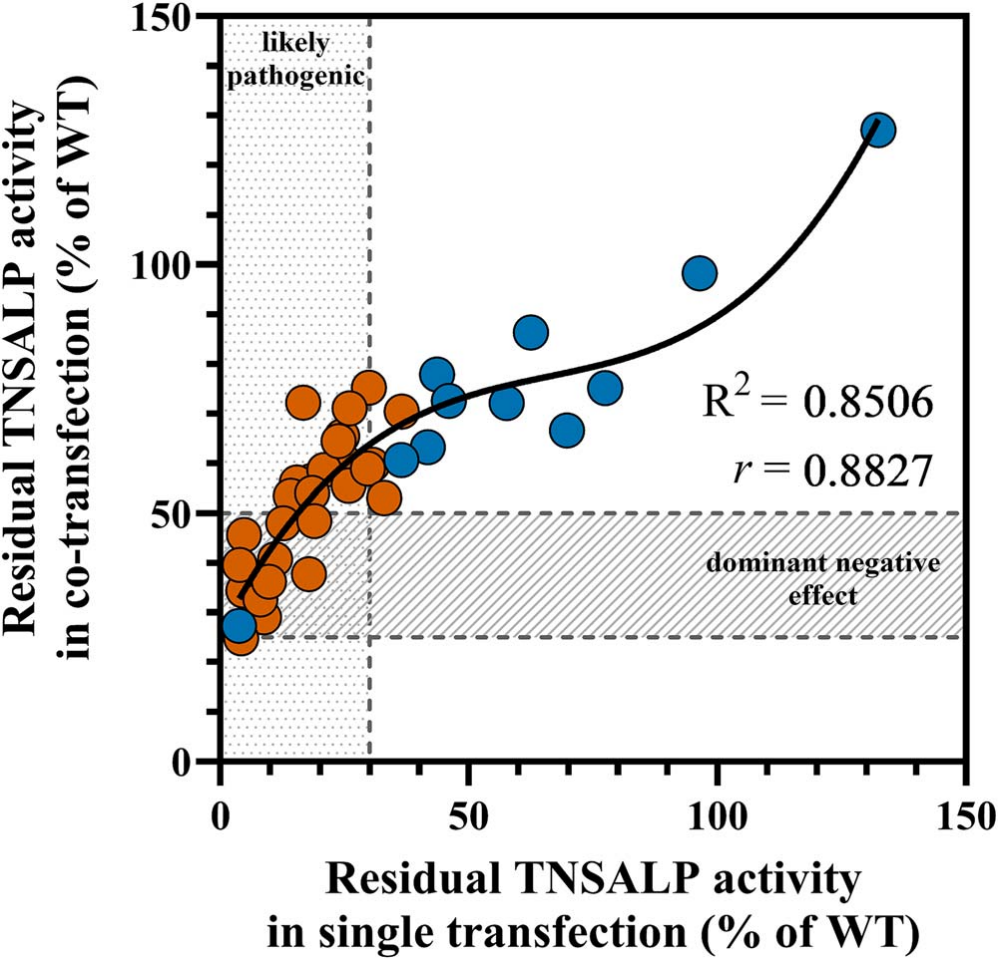
Correlation (*r*) and regression (*R*) analysis of TNSALP residual activities (% of WT) between single transfection and co-transfection for each variant tested. Blue dots represent variants reclassified as VUS after functional testing, while orange dots represent variants reclassified as likely pathogenic. Of 29 LP/P variants, 14 (48%) were dominant negative. Nonlinear regression analysis (*R*^2^) and Pearson correlation (*r*) of all *in vitro*tested variants (A; *n* = 40), only VUS-classified variants (B; *n* = 11), and only P/LP-classified variants (C; *n* = 29). Variants with ˂15% residual activity in single transfection show a DNE, while those with over 20% do not. Abbrevations: WT, wild type; LP, likely pathogenic; P, pathogenic; TNSALP, tissue-nonspecific alkaline phosphatase; VUS, variant of uncertain significance.

Next, we assessed the correlation between residual activity levels and DNE obtained from single and co-transfection models. As expected, among the 40 *ALPL*variants tested *in vitro*, a strong positive correlation was observed (*r* = 0.88, Figure 7); *ALPL*variants with lower residual activity in single transfection were more likely to exhibit a DNE in the co-transfection model (Figure 7). Specifically, 72% (29/40) of the *in vitro*tested variants demonstrated residual activity of ≤30% in the single transfection model, and 48% (14/29) of these variants were dominant negative (co-transfection residual activity between 25% and 50%). All pathogenic/likely pathogenic variants with residual activity below 15% and none with residual activity above 20% in single transfection showed a DNE.

### New genotypes and phenotypes for the JKU ALPL gene variant database

The genotypes and phenotypes (including asymptomatic states) associated with the newly reclassified variants were integrated with data from other sources, resulting in a total of 136 new genotypes and 118 associated phenotypes added to variants in the JKU ALPL gene variant database. All genotypes with associated phenotypes, whether submitted, extracted from deep literature mining, or identified using AI during the variant reclassification process, were included in the database along with specific variant information. Notably, the database now explicitly specifies the phenotype of heterozygous “asymptomatic carriers” of likely pathogenic or pathogenic mutations, reflecting findings from the limited evidence presented in the literature. Overall, the JKU ALPL gene variant database entries have grown by 21% since 2021.

## Discussion

To date, the ALPL gene variant consortium has conducted a meticulous evaluation and reclassification of 100 *ALPL*variants. This thorough process has resulted in the successful reclassification of 77% of the variants, while 23% remained VUS due to insufficient phenotypic data or conflicting evidence between the phenotype and *in vitro*functional studies. To date, ACMG/AMP criteria provide no guidance when evidence is conflicting. Among the 40 variants subjected to *in vitro*functional testing, 72% (*n* = 29) were reclassified as pathogenic or likely pathogenic, and 48% (14/29) exhibited a DNE. In this study, we identified that all variants with residual activity below 15%, but none with residual activity above 20%, displayed a DNE.

A thorough understanding of the type and localization of structural changes within TNSALP is essential for enhancing our comprehension of the impact of *ALPL*variants on protein stability, function, and hotspots contributing to HPP pathophysiology. However, it is important to note that the domain analysis is limited, as we cannot distinguish between the absence of protein and mutated protein in residual activity analysis. Thus, one of the aims of the project is to leverage the collected data to pinpoint hotspots where pathogenic/likely pathogenic and benign/likely benign variants tend to aggregate. To achieve this, the generated data was analyzed based on the type of variant, exon location, and protein domain. In our study, we found that variants such as frameshifts, in-frame insertion/deletions, splice site alterations, and nonsense variants have more adverse consequences as reported by others.^29^Our data shows that exon 12 exhibits the highest number of mutations, consistent with its second-highest frequency ranking of 14% (62 out of 452 recorded variants) in the JKU ALPL gene variant database. However, exon 12 is also the longest exon, providing more nucleotides and increasing the likelihood of mutations. Exclusively within exon 10 and exon 4, there reside pathogenic and likely pathogenic variants in alignment with the findings from the JKU ALPL gene variant database. We also demonstrate that *ALPL*variants located in the active site and homodimeric interface of TNSALP were more likely to result in a damaging effect than other domains, consistent with previous studies by Del Angel and Mornet.^29^^,^^30^Our study also illustrated that all DNE variants were missense. Of the 14 variants showing DNE *in vitro*functional studies, five were at the active site, four at the homodimeric interface, two in the crown domain, and the remaining in unspecified regions. This finding aligns with previous analyses, demonstrating that most variants exhibiting DNE are situated in the active site or its proximity, the crown domain, or the homodimeric interface.^31^^,^^32^Thus, these domains are crucial for protein activity.^4^^,^^30–32^Yu et al.^4^found that variants at the active site are pathogenic. Our data align with this, as all tested *ALPL*variants at the active site demonstrated ˂30% residual activity and DNE. Yu et al.^4^also observed that most variants at the homodimeric interface led to low expression and unstable TNSALP protein, with a few being less damaging. Our study revealed a similar pattern; of the 25 *ALPL*variants at the homodimeric interface, three were VUS, one was likely benign, and the rest were pathogenic/likely pathogenic, with residual activities ranging from 4% to 70%. Similarly, crown domain variants disrupt TNSALP dimerization^4^; in our data, five of seven variants in the crown domain were reclassified as likely pathogenic and two as VUS, showing that mutations in the crown domain affect TNSALP function. Identifying mutation hotspots and therapeutic targets for *ALPL*-related diseases is crucial for facilitating timely and accurate decision-making within the medical community. Although no hotspots have been identified to date, our ongoing research holds the potential to unveil them.

Twenty-nine of the 40 variants tested showed ˂30% residual activity, and 27/29 variants were not found in the Genome Aggregation Database (gnomAD; https://gnomad.broadinstitute.org). The remaining two variants, c.802T>C and c.707A>G, both had extremely low frequency (total) in gnomAD (v3.1.2) of ƒ = 0.000006575. All DNE variants were absent from gnomAD, aligning with the evolutionary pressure against variants with low enzymatic activity.^29^So far, our findings suggest that variants with low residual activity are more likely to be disease-causing, consistent with the published literature.^29^^,^^33^^,^^34^

Variant classification according to ACMG/AMP guidelines is based on strict, adapted criteria for *ALPL*, including variant frequency in population databases, functional data, and the clinical phenotype. Among the 100 reclassified variants, 23 were identified as VUS. Of these, 11 underwent *in vitro*testing. negligibleThe remaining variants were not selected for functional testing due to their failure to meet the criteria for likely pathogenic or pathogenic classification under ACMG standards. This decision was based on a combination of insufficient clinical evidence and resource considerations, as conducting functional testing on all variants would not be feasible within the scope of this study. For example, while variant c.176G>A exhibited low enzyme activity *in vitro*, this alone was insufficient for reclassification. It remained a VUS due to the absence of compelling clinical phenotypes, highlighting the importance of integrating detailed phenotyping in the classification process.

Conversely, variants c.1444C>A and c.1415A>G, with higher residual activities (33.1%, 36.1%), could be reclassified as likely pathogenic due to strong clinical evidence of HPP, which indicates that chosen thresholds (30%) will need constant adaptation to new evidence Although the ACMG criteria represent a well-established method for reclassifying variants, it is accompanied by a limitation: the absence of guidelines for variant classification in the presence of conflicting evidence. Given these learning points, we strongly encourage the global medical community to submit unreported phenotypes and genotypes to the ALPL gene variant project.

The variability in residual activity measurements in *in vitro*functional testing assays deserves some discussion points. We employed the same functional testing previously used by Del Angel and Mornet.^29^^,^^32^^,^^35^We validated the assay using thirteen controls, including all six known benign and seven pathogenic missense *ALPL*variants (Figure S2). Some tested benign variants exhibited higher residual activities, occasionally exceeding 100% of WT levels, with higher standard deviations compared to likely pathogenic variants, thereby widening the confidence interval. Overall, the specific residual activity percentage above 75% for benign variants is considered less critical for classification. Additionally, our enzymatic measurement technique shows increased variability in TNSALP activity as signal strength intensifies. The standard deviation is smaller when there is no signal or a very low signal compared to WT activity. A potential limitation of our study is that the *in vitro*reaction conditions, including the use of an alkaline buffer and pNPP as a substrate, may not accurately reflect the physiological environment in which human TNSALP functions. These conditions could affect the enzymatic activity of certain variants differently compared to their behavior *in vivo*, emphasizing the need for caution when extrapolating *in vitro*findings to clinical contexts. The amount of TNSALP generated post-transfection was not evaluated through methods such as Western blot analysis, due to limitations related to time and financial resources.

Throughout the reclassification process, a noteworthy observation is the large phenotypic variability associated with HPP, posing a diagnostic challenge. Traditional classifications often inadequately capture the dynamic nature of the disease and the overlapping symptoms among HPP subtypes as well as between HPP and other conditions. One of our primary objectives is to expand the spectrum of the HPP phenotype beyond traditional subtypes to improve clinical understanding. Genetic testing is crucial for precise diagnosis and has implications for initiation of therapy in the form of ERT (asfotase alfa, Strensiq®, Alexion Pharmaceuticals).^36^ERT is currently reserved for patients with a confirmed diagnosis of pediatric-onset HPP,^37^except in Japan where asfotase alfa is approved for all patients with HPP regardless of age of onset.^38^Clinical trials with a longer acting ERT molecule (ALXN1850)^39^for patients with confirmed HPP, regardless of age of onset, have been initiated. In this context, the JKU ALPL gene variant database serves as a valuable resource by providing information about *ALPL*variants and all associated genotypes and phenotypes, enabling clinicians and geneticists to provide informed guidance to patients and their families as well as ensuring early and correct diagnosis.

In conclusion, HPP is a rare disease exhibiting a wide phenotypic spectrum of severity, which necessitates comprehensive data collection. The ALPL gene variant consortium diligently collects and interprets all data in the JKU ALPL gene variant database. Here, we demonstrate that out of the first 100 reclassified variants, 73% were in the pathogenic and 4% in the benign spectrum, whereas 23% remained VUS. The consortium’s work has lead to the substantial expansion of information in the JKU ALPL gene variant database, with 21% more variants, 136 new genotypes and 118 associated phenotypes added since project initiation in 2021. This project enhances our genetic and clinical understanding of the disease spectrum, thus facilitating geneticists, researchers, and clinicians to grasp the intricacies of genetic variations and their related phenotypic traits. The accrued evidence on these newly reclassified *ALPL*variants will also provide valuable insights for healthcare providers, aiding in clinical management and genetic counseling.

## Supplementary Material

HPP_First_Results_SupplementaryMaterial_ziaf044

Origene_ziaf044

pSV-Beta-Galactosidase_ziaf044

## Data Availability

Data are available on the project website: https://ALPLmutationdatabase.jku.at/.
