# Supplementary material for: The ALPL gene variant project: results of the first 100 reclassified variants
Source: JBMR Plus. 2025 Mar 17;9(6):ziaf044. doi: 10.1093/jbmrpl/ziaf044 (PMC12083982; doi:10.1093/jbmrpl/ziaf044)
Supplement: HPP_First_Results_SupplementaryMaterial_ziaf044 [file hpp_first_results_supplementarymaterial_ziaf044.docx]

**Supplementary Table 1**: Specifications to ACMG/AMP variant classification criteria, for the classification of *ALPL* variants.

| **Criterion** | **Description** | **Specification** | **Additional considerations/comments** |
| --- | --- | --- | --- |
| **PVS1** | Null variant in a gene where loss of function (LOF)  is a known mechanism of disease | - Initiator codon variants will meet PVS1_Strong  - Nonsense and frameshift variants will meet PVS1 (Very Strong). If NMD is not predicted to occur, use PVS1_Moderate  - Variants affecting splice sites (+/- 2 position in intron) predicted to lead to out-of-frame exon skipping will meet PVS1_Strong. If exon skipping is in-frame or if there is a nearby cryptic splice site that may reconstitute in-frame splicing downgrade PVS1 accordingly. | -If PVS1 is applied, PM4 (protein length changing variant) will not be applied  - PS3 and PP3 should generally not be applied together with PVS1 |
| **PS1** | Same amino acid change as a previously established pathogenic variant regardless of nucleotide change | Apply moderate strength for likely pathogenic variants affecting the same codon. | Beware of changes that impact splicing rather than at the amino acid/protein level |
| **PS2** | De novo (both maternity and paternity confirmed) in a patient with the disease and no family history | Not applicable as de novo variants are rarely reported in *ALPL* |  |
| **PS3** | Well-established in vitro or in vivo functional studies supportive of a damaging effect | Strength depends on the number of controls used in the functional study. Our functional study has 13 controls (seven pathogenic and six benign). According to the ClinGen recommendations (PMID: 31892348) we can only apply moderate strength to this criterion. Since there are no more known benign missense variants to date, it is currently not possible to use this criterion as strong. | Thresholds of ALP activity:  -Activity up to 30%: apply PS3_moderate  -Activity between 31 and 50%: apply PS3_supporting |
| **PS4** | The prevalence of the variant in affected individuals is significantly increased compared with the prevalence in controls | Not applicable: There are no case-control studies for HPP |  |
| **PM1** | Located in a mutational hot spot and/or critical and well-established functional domain without benign variation | - Apply PM1 for variants affecting residues in the active site of the enzyme, as well as cysteine residues (for the role of cysteine residues in *ALPL* stability see PMID: 22266140) | Active site amino acid residues: 60, 109-111, 171, 173, 184, 187, 332, 337, 341, 378-379, 381, 454 |
| **PM2** | Low frequency in population databases | According to ClinGen recommendations apply only as supporting |  |
| **PM3** | Detected in trans with a pathogenic variant | Apply according to the ClinGen point-based system (strength depending on the pathogenicity and the phase of the second variant). | See: https://clinicalgenome.org/working-groups/sequence-variant-interpretation/ |
| **PM4** | Protein length changes as a result of in-frame deletions/insertions in a non-repeat region or stop-loss variants | -Downgrade to PM4_Supporting for single amino acid deletions and insertions. If the deletion affects a cysteine residue or more than one amino acid, use moderate.  -For in-frame deletions of one or more exons, use PVS1 |  |
| **PM5** | Novel missense change at an amino acid residue where a different missense change determined to be pathogenic has been seen before | -If the other variant is Pathogenic, use PM5.  -If the other variant is Likely Pathogenic, use PM5_Supporting | Beware of changes that impact splicing rather than at the amino acid/protein level |
| **PM6** | Assumed de novo but without confirmation of paternity and maternity | Not applicable (see PS2) |  |
| **PP1** | Cosegregation with disease in multiple affected family members in a gene definitively known to cause the disease | Use at supporting strength in case family data is available |  |
| **PP2** | Missense variant in a gene that has a low rate of benign missense variation and where missense variants are a common mechanism of disease | Apply for all missense variants at supporting strength |  |
| **PP3** | Multiple lines of computational evidence support a deleterious effect on the gene or gene product | Use in supporting strength if:  -REVEL score ≥ 0.70  -SpliceAI shows a splice change (score of >0.2) |  |
| **PP4** | Phenotype specific for disease with single genetic etiology | Use phenotype scoring algorithm published in our previous work: PMID: 37898381 |  |
| **PP5** | Reputable source recently reports variant as pathogenic, but the evidence is not available to the laboratory to perform an independent evaluation | According to ClinGen recommendations, this criterion is not used | See also: PMID: 29543229 |

**Supplementary Table 2:** The *ALPL* variants tested in the JKU lab with their reclassification, single transfection mean residual activity (%), and co-transfection mean residual activity (%) including the presence of a dominant negative effect (DNE).

|  | **Variant** | **Single Transfection Residual Activity %** | **Co-transfection Residual Activity %** | **Reclassification** |
| --- | --- | --- | --- | --- |
| 1 | c.41T>C | 16.7 | 72.2 | Likely Pathogenic |
| 2 | c.69-74del | 15.4 | 56.1 | Likely Pathogenic |
| 3 | c.106A>C | 14.6 | 49.7 | Likely Pathogenic |
| 4 | c.140A>T | 23.8 | 64.4 | Likely Pathogenic |
| 5 | c.146A>T | 24.8 | 60.4 | Likely Pathogenic |
| 6 | c.176G>A | 3.8 | 27.3 | VUS |
| 7 | c.178G>C | 17.8 | 37.7 | Likely Pathogenic |
| 8 | c.194C>A | 20.9 | 58.6 | Likely Pathogenic |
| 9 | c.206C>T | 69.7 | 66.6 | VUS |
| 10 | c.214A>G | 25.8 | 55.5 | Likely Pathogenic |
| 11 | c.244G>A | 5.4 | 37.1 | Likely Pathogenic |
| 12 | c.286G>C | 14.2 | 53.4 | Likely Pathogenic |
| 13 | c.295A>G | 4.8 | 45.6 | Likely Pathogenic |
| 14 | c.302A>G | 8.8 | 29 | Likely Pathogenic |
| 15 | c.319G>A | 29.9 | 75.2 | Likely Pathogenic |
| 16 | c.361G>A | 30.6 | 59.7 | Likely Pathogenic |
| 17 | c.466G>T | 77.4 | 75.1 | VUS |
| 18 | c.511C>G | 12.7 | 48 | Likely Pathogenic |
| 19 | c.538C>A | 96.4 | 98.2 | VUS |
| 20 | c.601T>C | 18.5 | 54 | Likely Pathogenic |
| 21 | c.625A>T | 41.7 | 63.2 | VUS |
| 22 | c.707A>G | 29.7 | 58.9 | Likely Pathogenic |
| 23 | c.715G>T | 132.4 | 127.1 | VUS |
| 24 | c.802T>C | 24.5 | 65.4 | Likely Pathogenic |
| 25 | c.967A>G | 43.6 | 77.8 | VUS |
| 26 | c.1000G>A | 11 | 40.7 | Likely Pathogenic |
| 27 | c.1034C>T | 4.6 | 34.4 | Likely Pathogenic |
| 28 | c.1156G>C | 18.3 | 56.4 | Likely Pathogenic |
| 29 | c.1157G>A | 8.1 | 32.6 | Likely Pathogenic |
| 30 | c.1213A>C | 9.8 | 36.2 | Likely Pathogenic |
| 31 | c.1225C>G | 36.4 | 60.6 | VUS |
| 32 | c.1225C>T | 46 | 72.5 | VUS |
| 33 | c.1247G>A | 4.2 | 24.8 | Likely Pathogenic |
| 34 | c.1327G>T | 26 | 71 | Likely Pathogenic |
| 35 | c.1331A>G | 18.9 | 48.4 | Likely Pathogenic |
| 36 | c.1376T>C | 3.9 | 39.6 | Likely Pathogenic |
| 37 | c.1379C>T | 57.6 | 72.1 | VUS |
| 38 | c.1415A>G | 36.5 | 70.4 | Likely Pathogenic |
| 39 | c.1444C>A | 33 | 53 | Likely Pathogenic |
| 40 | c.1487A>G | 62.5 | 86.3 | VUS |


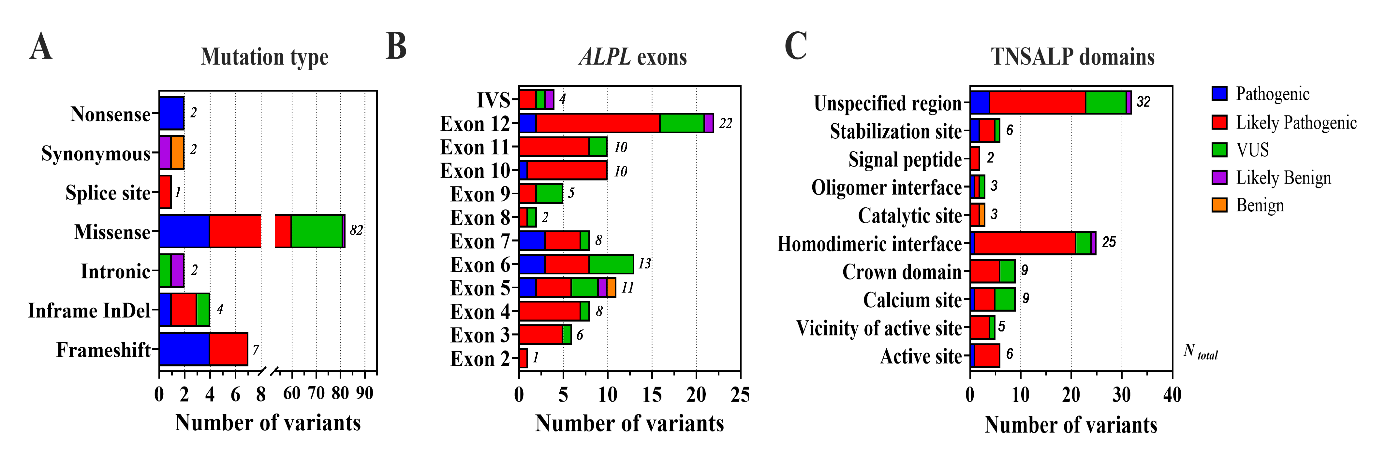
**Supplementary Figure 1: Overview of the reclassified VUS according to variant type, location, and protein domain**. Data is represented as grouped bar graphs. (A) shows the number of reclassified variants with specific pathogenicity for each variant type. (B) shows the number of variants with specific pathogenicity at each exon of the ALPL gene. (C) shows the number of variants with specific pathogenicity in the different domains of the ALPL protein. P, pathogenic; LP, likely pathogenic; VUS, a variant of uncertain significance; LB, Likely benign; B, Benign.


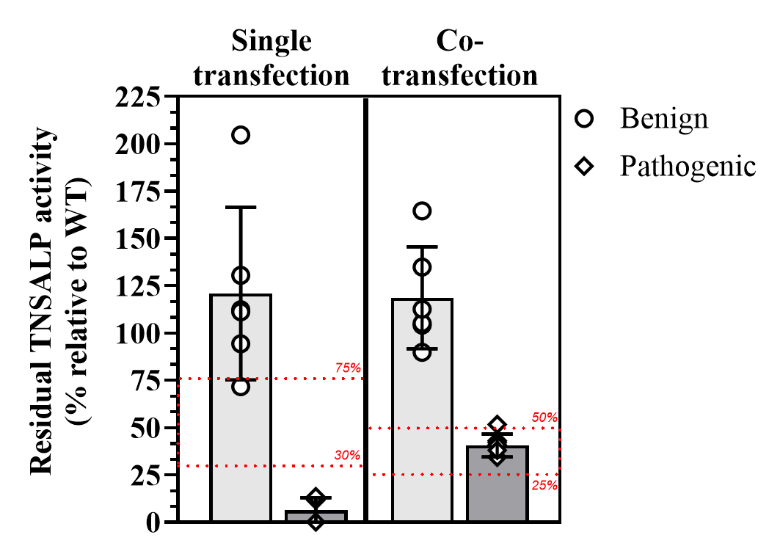


**Supplementary Figure 2: Functional Assay Validation.** The bar graph shows the residual TNSALP activities of six known benign and seven known pathogenic (all DNE) variants used to validate the functional testing assay.
