# Supplementary material for: The ALPL gene variant project: results of the first 100 reclassified variants
Source: JBMR Plus. 2025 Mar 17;9(6):ziaf044. doi: 10.1093/jbmrpl/ziaf044 (PMC12083982; doi:10.1093/jbmrpl/ziaf044)
Supplement: Origene_ziaf044 [file origene_ziaf044.pdf]

OriGene Technologies, Inc.
9620 Medical Center Drive, Ste 200
Rockville, MD 20850, US
Phone: +1-888-267-4436
https://www.origene.com
techsupport@origene.com
EU: info-de@origene.com
CN: techsupport@origene.cn

Product datasheet for RC205692

Alkaline Phosphatase (ALPL) (NM_000478) Human Tagged ORF Clone

Product data:

**Product Type:**	Expression Plasmids
**Product Name:**	Alkaline Phosphatase (ALPL) (NM_000478) Human Tagged ORF Clone
**Tag:**	Myc-DDK
**Symbol:**	Alkaline Phosphatase
**Synonyms:**	AP-TNAP; APTNAP; HOPS; HPPA; HPPC; HPPI; HPPO; TNALP; TNAP; TNSALP
**Mammalian Cell Selection:**	Neomycin
**Vector:**	pCMV6-Entry (PS100001)
**E. coli Selection:**	Kanamycin (25 ug/mL)

View online »

This product is to be used for laboratory only. Not for diagnostic or therapeutic use.
©2024 OriGene Technologies, Inc., 9620 Medical Center Drive, Ste 200, Rockville, MD 20850, US

ORF Nucleotide Sequence:

>RC205692 ORF sequence
Red=Cloning site Blue=ORF Green=Tags(s)

TTTTGTAATACGACTCACTATAGGGCGGCCGGGAATTCGTCGACTGGATCCGGTACCGAGGAGATCTGCC
GCCGCGATCGCC

ATGATTTCACCATTCTTAGTACTGGCCATTGGCACCTGCCTTACTAACTCCTTAGTGCCAGAGAAAGAGA
AAGACCCCAAGTACTGGCGAGACCAAGCGCAAGAGACACTGAAATATGCCCTGGAGCTTCAGAAGCTCAA
CACCAACGTGGCTAAGAATGTCATCATGTTCCTGGGAGATGGGATGGGTGTCTCCACAGTGACGGCTGCC
CGCATCCTCAAGGGTCAGCTCCACCACAACCCTGGGGAGGAGACCAGGCTGGAGATGGACAAGTTCCCCT
TCGTGGCCCTCTCCAAGACGTACAACACCAATGCCCAGGTCCCTGACAGCGCCGGCACCGCCACCGCCTA
CCTGTGTGGGGTGAAGGCCAATGAGGGCACCGTGGGGGTAAGCGCAGCCACTGAGCGTTCCCGGTGCAAC
ACCACCCAGGGGAACGAGGTCACCTCCATCCTGCGCTGGGCCAAGGACGCTGGGAAATCTGTGGGCATTG
TGACCACCACGAGAGTGAACCATGCCACCCCCAGCGCCGCCTACGCCCACTCGGCTGACCGGGACTGGTA
CTCAGACAACGAGATGCCCCCTGAGGCCTTGAGCCAGGGCTGTAAGGACATCGCCTACCAGCTCATGCAT
AACATCAGGGACATTGACGTGATCATGGGGGGTGGCCGGAAATACATGTACCCCAAGAATAAAACTGATG
TGGAGTATGAGAGTGACGAGAAAGCCAGGGGCACGAGGCTGGACGGCCTGGACCTCGTTGACACCTGGAA
GAGCTTCAAACCGAGATACAAGCACTCCCACTTCATCTGGAACCGCACGGAACTCCTGACCCTTGACCCC
CACAATGTGGACTACCTATTGGGTCTCTTCGAGCCAGGGGACATGCAGTACGAGCTGAACAGGAACAACG
TGACGGACCCGTCACTCTCCGAGATGGTGGTGGTGGCCATCCAGATCCTGCGGAAGAACCCCAAAGGCTT
CTTCTTGCTGGTGGAAGGAGGCAGAATTGACCACGGGCACCATGAAGGAAAAGCCAAGCAGGCCCTGCAT
GAGGCGGTGGAGATGGACCGGGCCATCGGGCAGGCAGGCAGCTTGACCTCCTCGGAAGACACTCTGACCG
TGGTCACTGCGGACCATTCCCACGTCTTCACATTTGGTGGATACACCCCCCGTGGCAACTCTATCTTTGG
TCTGGCCCCCATGCTGAGTGACACAGACAAGAAGCCCTTCACTGCCATCCTGTATGGCAATGGGCCTGGC
TACAAGGTGGTGGGCGGTGAACGAGAGAATGTCTCCATGGTGGACTATGCTCACAACAACTACCAGGCGC
AGTCTGCTGTGCCCCTGCGCCACGAGACCCACGGCGGGGAGGACGTGGCCGTCTTCTCCAAGGGCCCCAT
GGCGCACCTGCTGCACGGCGTCCACGAGCAGAACTACGTCCCCCACGTGATGGCGTATGCAGCCTGCATC
GGGGCCAACCTCGGCCACTGTGCTCCTGCCAGCTCGGCAGGCAGCCTTGCTGCAGGCCCCCTGCTGCTCG
CGCTGGCCCTCTACCCCCTGAGCGTCCTGTTC

ACGCGTACGCGGCCGCTCGAGCAGAAACTCATCTCAGAAGAGGATCTGGCAGCAAATGATATCCTGGATT
ACAAGGATGACGACGATAAGGTTTAA

Protein Sequence:

>RC205692 protein sequence
Red=Cloning site Green=Tags(s)

MISPFLVLAIGTCLTNSLVPEKEKDPKYWRDQAQETLKYALELQKLNTNVAKNVIMFLGDGMGVSTVTAA
RILKGQLHHNPGEETRLEMDKFPFVALSKTYNTNAQVPDSAGTATAYLCGVKANEGTVGVSAATERSRCN
TTQGNEVTSILRWAKDAGKSVGIVTTTRVNHATPSAAYAHSADRDWYSDNEMPPEALSQGCKDIAYQLMH
NIRDIDVIMGGGRKYMYPKNKTDVEYESDEKARGTRLDGLDLVDTWKSFKPRYKHSHFIWNRTELLTLDP
HNVDYLLGLFEPGDMQYELNRNNVTDPSLSEMVVVAIQILRKNPKGFFLLVEGGRIDHGHHEGKAKQALH
EAVEMDRAIGQAGSLTSSEDTLTVVTADHSHVFTFGGYTPRGNSIFGLAPMLSDTDKKPFTAILYGNGPG
YKVVGGERENVSMVDYAHNNYQAQSAVPLRHETHGGEDVAVFSKGPMAHLLHGVHEQNYVPHVMAYAACI
GANLGHCAPASSAGSLAAGPLLLALALYPLSVLF

TRTRPLEQKLISEEDLAANDILDYKDDDDKV

Chromatograms: https://cdn.origene.com/chromatograms/mk6199_f10.zip

Restriction Sites: SgfI-MluI

This product is to be used for laboratory only. Not for diagnostic or therapeutic use.
©2024 OriGene Technologies, Inc., 9620 Medical Center Drive, Ste 200, Rockville, MD 20850, US

Cloning Scheme:

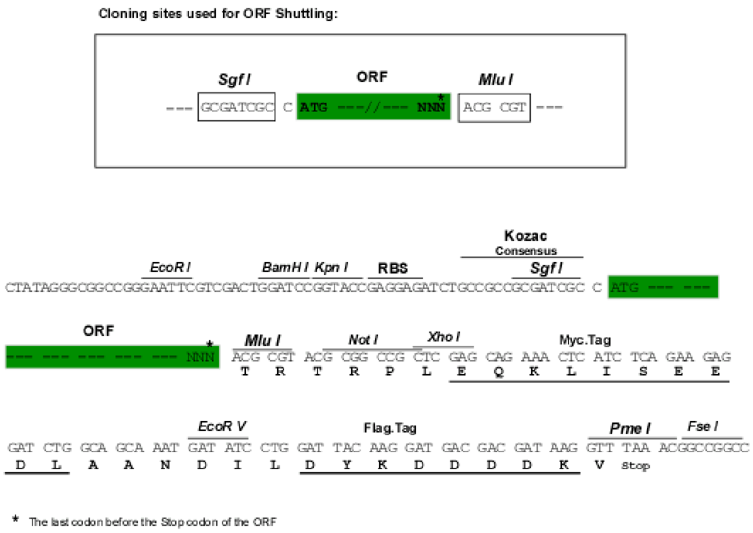

ACCN: NM_000478

ORF Size: 1572 bp

OTI Disclaimer: The molecular sequence of this clone aligns with the gene accession number as a point of reference only. However, individual transcript sequences of the same gene can differ through naturally occurring variations (e.g. polymorphisms), each with its own valid existence. This clone is substantially in agreement with the reference, but a complete review of all prevailing variants is recommended prior to use. More info

OTI Annotation: This clone was engineered to express the complete ORF with an expression tag. Expression varies depending on the nature of the gene.

Components: The ORF clone is ion-exchange column purified and shipped in a 2D barcoded Matrix tube containing 10ug of transfection-ready, dried plasmid DNA (reconstitute with 100 ul of water).

Reconstitution Method:
1. Centrifuge at 5,000xg for 5min.
2. Carefully open the tube and add 100ul of sterile water to dissolve the DNA.
3. Close the tube and incubate for 10 minutes at room temperature.
4. Briefly vortex the tube and then do a quick spin (less than 5000xg) to concentrate the liquid at the bottom.
5. Store the suspended plasmid at -20°C. The DNA is stable for at least one year from date of shipping when stored at -20°C.

Note: Plasmids are not sterile. For experiments where strict sterility is required, filtration with 0.22um filter is required.

RefSeq: NM_000478.5

RefSeq Size: 2606 bp

RefSeq ORF: 1575 bp

Locus ID: 249

This product is to be used for laboratory only. Not for diagnostic or therapeutic use.
©2024 OriGene Technologies, Inc., 9620 Medical Center Drive, Ste 200, Rockville, MD 20850, US

UniProt ID: P05186

Cytogenetics: 1p36.12

Domains: alk_phosphatase

Protein Families: Druggable Genome

Protein Pathways: Folate biosynthesis, Metabolic pathways

MW: 57.3 kDa

Gene Summary: This gene encodes a member of the alkaline phosphatase family of proteins. There are at least four distinct but related alkaline phosphatases: intestinal, placental, placental-like, and liver/bone/kidney (tissue non-specific). The first three are located together on chromosome 2, while the tissue non-specific form is located on chromosome 1. The product of this gene is a membrane bound glycosylated enzyme that is not expressed in any particular tissue and is, therefore, referred to as the tissue-nonspecific form of the enzyme. Alternative splicing results in multiple transcript variants, at least one of which encodes a preproprotein that is proteolytically processed to generate the mature enzyme. This enzyme may play a role in bone mineralization. Mutations in this gene have been linked to hypophosphatasia, a disorder that is characterized by hypercalcemia and skeletal defects. [provided by RefSeq, Oct 2015]

Product images:

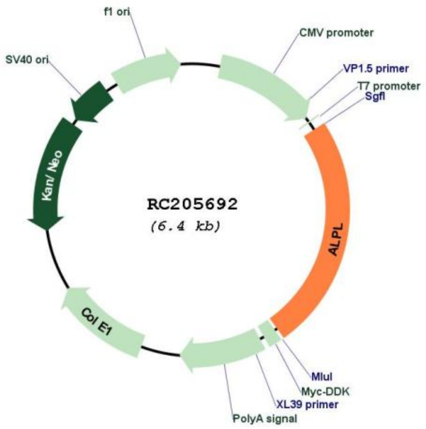

Circular map for RC205692

This product is to be used for laboratory only. Not for diagnostic or therapeutic use.
©2024 OriGene Technologies, Inc., 9620 Medical Center Drive, Ste 200, Rockville, MD 20850, US

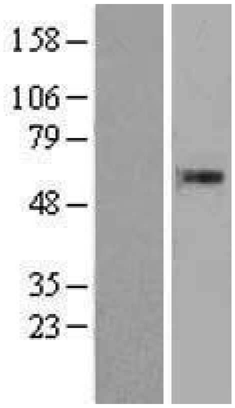

Western blot validation of overexpression lysate (Cat# [LY426804]) using anti-DDK antibody (Cat# [TA50011-100]). Left: Cell lysates from un-transfected HEK293T cells; Right: Cell lysates from HEK293T cells transfected with [RC225898] using transfection reagent MegaTran 2.0 (Cat# [TT210002]).

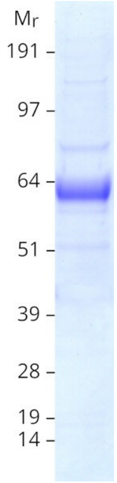

Coomassie blue staining of purified ALPL protein (Cat# [TP305692]). The protein was produced from HEK293T cells transfected with ALPL cDNA clone (Cat# RC205692) using MegaTran 2.0 (Cat# [TT210002]).

©2024 OriGene Technologies, Inc., 9620 Medical Center Drive, Ste 200, Rockville, MD 20850, US
