# Supplementary material for: The ALPL gene variant project: results of the first 100 reclassified variants
Source: JBMR Plus. 2025 Mar 17;9(6):ziaf044. doi: 10.1093/jbmrpl/ziaf044 (PMC12083982; doi:10.1093/jbmrpl/ziaf044)
Supplement: pSV-Beta-Galactosidase_ziaf044 [file psv-beta-galactosidase_ziaf044.pdf]

Promega

Technical Bulletin

pSV-β-Galactosidase Control Vector

INSTRUCTIONS FOR USE OF PRODUCT E1081.

PRINTED IN USA.
Revised 9/06

www.promega.com

AF9TB094 0906TB094

Part# TB094

Promega

pSV-β-Galactosidase Control Vector

All technical literature is available on the Internet at: www.promega.com/tbs/
Please visit the web site to verify that you are using the most current version of this Technical Bulletin. Please contact Promega Technical Services if you have questions on use of this system. E-mail: techserv@promega.com

I. Description 1
II. Product Components and Storage Conditions 2
III. General Usage Considerations 2
IV. pSV-β-Galactosidase Control Vector Circle Map 3
V. pSV-β-Galactosidase Control Vector Restriction Sites 4
VI. References 6
VII. Related Products 7

I. Description

The pSV-β-Galactosidase Control Vector is designed as a positive control vector for monitoring transfection efficiencies of mammalian cells. The SV40 early promoter and enhancer drive transcription of the bacterial *lacZ* gene, which in turn, is translated into the β-galactosidase enzyme. β-galactosidase is an excellent reporter enzyme (1,2) that can be assayed quickly and directly in cell extracts using spectrophotometric, fluorescent or chemiluminescent assays (3,4). This reporter enzyme is also widely used for in situ histochemical analysis using the substrate X-Gal (5).

The pSV-β-Galactosidase Control Vector can be co-transfected with your DNA of interest. For example, co-transfection with firefly luciferase gene vectors provide cell extracts that can be assayed for both luciferase and β-galactosidase activities. In this manner, the pSV-β-Galactosidase Vector acts as an internal control for transient expression assays. A negative control extract, prepared from mock-transfected cells, should also be assayed for the presence of endogenous β-galactosidase activity in cultured cells (2). In addition, co-transfection with chloramphenicol acetyltransferse reporter gene vectors (e.g., pCAT®3 Vectors) permits assaying for both CAT and β-galactosidase activities.

I. Description (continued)

The pSV-β-Galactosidase Vector is a modification of pRSV-βGAL (6) with SV40 and pUC18 sequences substituted for RSV and pBR322 sequences. The pSV-β-Galactosidase Vector will express β-galactosidase in *E. coli* due to the presence of the *E. coli gpt* promoter located upstream of the *lac*Z gene (1). Colonies of *E. coli* containing the pSV-β-Galactosidase Vector will appear blue when plated on media containing X-gal.

II. Product Components and Storage Conditions

Product	Size	Cat.#
pSV-β-Galactosidase Control Vector	20μg	E1081

Storage Conditions: Store the pSV-β-Galactosidase Control Vector at –20°C.

III. General Usage Considerations

The recommended amount of pSV-β-Galactosidase Control Vector to use for transfection of cells (60 or 100mm dish) is 5–10μg. The optimal amount of plasmid DNA will be determined by the efficiency of transfection, which is dependent upon the particular cell line and transfection protocol.

Several methods can be used to prepare cell extracts to be assayed for β-galactosidase activity. Reporter Lysis Buffer, supplied with the β-Galactosidase Enzyme Assay System with Reporter Lysis Buffer (Cat.# E2000), or available separately (Cat.# E3971) allows β-galactosidase, chloramphenicol acetyltransferase (CAT) and luciferase assays to be performed from the same cell extract. In cell lines that we have tested, β-galactosidase activity in the Reporter Lysis Buffer is significantly higher than activity in extracts prepared with the freeze-thaw method. Complete instructions are supplied with the buffer.

For β-galactosidase assays not using the Reporter Lysis Buffer, we recommend the protocols found in the *Protocols and Applications Guide* chapter on Bioluminescence Reporters (7).

IV. pSV-β-Galactosidase Control Vector Circle Map

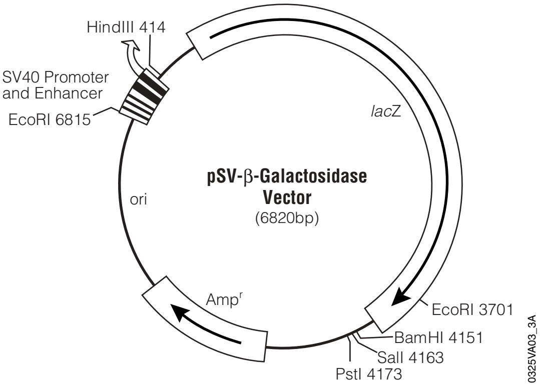

Figure 1. pSV-β-Galactosidase Control Vector circle map and sequence reference points.

Sequence reference points:

SV40 early promoter and enhancer segment	1–419
Transcription start sites	354, 360, 365
gpt promoter (–10 region)	428–433
Possible start codons (ATG)	500, 530, 569
operon sequences	709–4020
*lac*Z start site	710
*lac*Z stop site (TAA)	3755
*lac*Y	3809–4011
SV40 small T antigen	4021–4156
β-lactamase (Amp^r) coding region	4784–5644

Note: The *lac*Z coding region in this vector starts with the 7th amino acid of the wildtype *lac*Z gene.

V. pSV-β-Galactosidase Control Vector Restriction Sites

The following restriction enzyme tables were constructed using DNASTAR® sequence analysis software. Please note that we have not verified this information by restriction digestion with each enzyme listed. The location given specifies the 3´ end of the cut DNA (the base to the left of the cut site). For more information on the cut sites of these enzymes, or if you identify a discrepancy, please contact your local Promega Branch or Distributor. In the U.S., contact Promega Technical Services at 800-356-9526. Vector sequences are available in the GenBank® database (GenBank®/EMBL Accession Number **X65335**) and on the Internet at: **www.promega.com/vectors/**

Table 1. Restriction Enzymes That Cut the pSV-β-Galactosidase Control Vector Between 1 and 5 Times.

Enzyme	# of Sites	Location
**AatII**	2	1320, 4652
**AccB7I**	2	1942, 2308
**AccI**	2	3461, 4164
**Acc65I**	2	48, 619
**AgeI**	1	622
**Alw44I**	5	3042, 3157, 4402, 4899, 6145
AlwNI	4	2407, 2832, 3583, 6050
**AvaI**	2	2035, 3478
**AvaII**	3	2238, 5207, 5429
AvrII	1	398
**BamHI**	1	4151
**BanII**	2	2637, 3686
BbeI	1	4348
BbsI	3	639, 2214, 3466
**BbuI**	3	146, 218, 4179
**BclI**	1	2043
**BglI**	5	351, 849, 2970, 4335, 5453
BlpI	1	3710
Bpu1102I	1	3710
BsaI	1	5505
BsaAI	1	548
BsaBI	1	2028
**BsaMI**	1	4101
BsmI	1	4101
BspHI	3	4626, 4731, 5739
BspMI	3	1471, 3343, 4176
**BssHII**	1	2195
BssSI	4	1754, 4595, 4902, 6286
Bst1107I	1	3462
**BstXI**	2	2917, 3534
**Bsu36I**	1	921
Cfr10I	4	622, 1984, 3716, 5486
**ClaI**	2	702, 1521
**DraI**	4	3819, 4993, 5685, 5704
DraII	1	4591
DraIII	1	1887
DrdI	3	2806, 4488, 6357
EaeI	5	1168, 2166, 4191, 5178, 6620
EarI	4	798, 4284, 4772, 6576
**EclHKI**	1	5571
**Eco47III**	1	2532
Eco81I	1	921
**EcoICRI**	1	2635
**EcoRI**	2	3701, 6814
**EcoRV**	1	1810
EheI	1	4346
FspI	3	839, 4325, 5348
**HincII**	4	1123, 1747, 3575, 4165

Table 1. Restriction Enzymes That Cut the pSV-β-Galactosidase Control Vector Between 1 and 5 Times (continued).

Enzyme	# of Sites	Location
HindII	4	1123, 1747, 3575, 4165
**HindIII**	1	414
**HpaI**	2	1123, 1747
KasI	1	4344
**KpnI**	2	52, 623
**MluI**	3	1999, 2779, 3204
**NarI**	1	4345
**NcoI**	2	9, 305
**NdeI**	2	3654, 4397
**NsiI**	2	148, 220
PflMI	2	1942, 2308
PinAI	1	622
PleI	5	3668, 4169, 5580, 6083, 6568
Ppu10I	2	144, 216

Enzyme	# of Sites	Location
**PstI**	1	4173
**SacI**	1	2637
**SalI**	1	4163
**ScaI**	1	5090
**SfiI**	1	351
**SinI**	3	2238, 5207, 5429
**SphI**	3	146, 218, 4179
SplI	1	3468
Sse8387I	1	4173
**SspI**	2	1927, 4766
**StuI**	1	397
**StyI**	3	9, 305, 398
**VspI**	3	5396, 6631, 6690
**XbaI**	1	4157
XcmI	3	2914, 3659, 3992
**XmnI**	1	4971

Table 2. Restriction Enzymes That Do Not Cut the pSV-β-Galactosidase Control Vector.

**AccIII**	**Bst98I**	FseI	PmeI	SgrAI
AflII	**BstEII**	**I-PpoI**	PmlI	**SmaI**
**ApaI**	**BstZI**	**NaeI**	PpuMI	**SnaBI**
AscI	**CspI**	**NgoMIV**	PshAI	**SpeI**
**BalI**	**Csp45I**	**NheI**	Psp5II	SrfI
BbrPI	EagI	**NotI**	PspAI	SwaI
**BglII**	**Eco52I**	**NruI**	RsrII	**Tth111I**
Bsp120I	Eco72I	PacI	**SacII**	**XhoI**
BsrGI	EcoNI	PaeR7I	**SgfI**	**XmaI**

Note: The enzymes listed in boldface type are available from Promega.

V. pSV-β-Galactosidase Control Vector Restriction Sites (continued)

Table 3. Restriction Enzymes That Cut the pSV-β-Galactosidase Control Vector Six or More Times.

AciI	BsrI	**HaeII**	**MboI**	**PvuII**
AcyI	**BsrSI**	**HaeIII**	**MboII**	**RsaI**
AflIII	Bst71I	HgaI	MnlI	**Sau3AI**
**AluI**	**BstOI**	**HhaI**	MseI	Sau96I
**Alw26I**	BstUI	**HinfI**	**MspI**	ScrFI
AspHI	**CfoI**	**HpaII**	**MspA1I**	SfaNI
**BanI**	**DdeI**	HphI	**NciI**	**TaqI**
BbvI	**DpnI**	**Hsp92I**	**NdeII**	TfiI
BsaOI	DpnII	**Hsp92II**	NlaIII	**Tru9I**
BsaHI	DsaI	MaeI	NlaIV	**XhoII**
BsaJI	Fnu4HI	MaeII	NspI	
**Bsp1286I**	**FokI**	MaeIII	**PvuI**	

Note: The enzymes listed in boldface type are available from Promega.

VI. References

1. Hall, C.V. *et al.* (1983) Expression and regulation of *Escherichia coli lacZ* gene fusions in mammalian cells. *J. Mol. Appl. Genet.* **2**, 101–9.

2. Rosenthal, N. (1987) Identification of regulatory elements of cloned genes with functional assays. *Methods Enzymol.* **152**, 704–20.

3. Silhavy, T. *et al.* (1984) In: *Experiments with Gene Fusions*, Cold Spring Harbor Laboratory, Cold Spring Harbor, New York, 266.

4. Schenborn, E. and Groskreutz, D. (1999) Reporter gene vectors and assays. *Mol. Biotechnol.* **13**, 29–44.

5. Lim, K. and Chae, C.B. (1989) A simple assay for DNA transfection by incubation of the cells in culture dishes with substrates for β-galactosidase. *Biotechniques* **7**, 576–9.

6. Edlund, T. *et al.* (1985) Cell-specific expression of the rat insulin gene: Evidence for role of two distinct 5´ flanking elements. *Science* **230**, 912–6.

7. *Protocols and Applications Guide*, Online Edition. (2006) Promega Corporation.

VII. Related Products

Product	Size	Cat.#
Beta-Glo® Assay System	10ml	E4720
	100ml	E4740
	10 × 100ml	E4780
β-Galactosidase Enzyme Assay System with Reporter Lysis Buffer	65 assays	E2000
Reporter Lysis Buffer	30ml	E3971

© 1991–2006 Promega Corporation. All Rights Reserved.

Beta-Glo and pCAT are registered trademarks of Promega Corporation.

DNASTAR is a registered trademark of DNASTAR, Inc. GenBank is a registered trademark of the U.S. Department of Health and Human Services.

Products may be covered by pending or issued patents or may have certain limitations. Please visit our Web site for more information.

All prices and specifications are subject to change without prior notice.

Product claims are subject to change. Please contact Promega Technical Services or access the Promega online catalog for the most up-to-date information on Promega products.
